# Assessment of melatonergics in prevention of delirium in critically ill patients: A protocol for systematic review and meta-analysis

**DOI:** 10.1097/MD.0000000000018700

**Published:** 2020-01-10

**Authors:** Yibing Zhu, Zhiming Jiang, Huibin Huang, Yang Wang, Linlin Zhang, Chao Ren, Yongming Yao, Wei Li, Bin Du, Xiuming Xi

**Affiliations:** a Medical Research and Biometrics Center, Fuwai Hospital, National Center for Cardiovascular Diseases, Chinese Academy of Medical Sciences and Peking Union Medical College; b Department of Critical Care Medicine, Fuxing Hospital, Capital Medical University, Beijing; c Department of Critical Care Medicine, the First Affiliated Hospital of Shandong First Medical University, Jinan, Shandong; d Medical ICU, Peking Union Medical College Hospital, Peking Union Medical College and Chinese Academy of Medical Sciences; e Department of Critical Care Medicine, Beijing Tsinghua Chang Gung Hospital; f Department of Critical Care Medicine, Beijing Tiantan Hospital, Capital Medical University, Beijing; g School of Medicine, Nankai University, Tianjin; h Trauma Research Center, First Hospital Affiliated to the Chinese PLA General Hospital; i State Key Laboratory of Kidney Disease, the Chinese PLA General Hospital, Beijing, China.

**Keywords:** critical care medicine, delirium, melatonin, prevention, systematic review

## Abstract

**Background:**

Delirium is a commonly occurred complication in the critically ill. Melatonin is an endogenous hormone exerting multiple biological effects, mainly in regulating diurnal rhythms, also in inflammatory process and immune response. We aimed to assess the efficacy of exogenous melatonergics in prevention of delirium.

**Methods:**

PubMed, Cochrane Library, and Embase will be searched to identify randomized controlled trials published from 1960 to April 2019. Critically ill adult patients administrated with melatonergics will be included. The primary outcome measure will be the incidence of delirium. The secondary outcome measure will be the length of stay in intensive care unit. The pooled effects of dichotomous outcomes will be analyzed as risk ratio, and that of continuous outcomes will be analyzed using weighted mean difference. Subgroup and sensitivity analyses will be conducted. Funnel plots and/or Egger test will be done for the examination of publication bias. The quality of evidence resulting from this study will be evaluated using the GRADE methodology. Trial sequential analysis (TSA) will be done to test whether the evidence in our meta-analysis is reliable and conclusive.

**Result:**

The evidence to date of the melatonergics in prevention of delirium will be systematically reviewed and meta-analyzed with the GRADE level reported and TSA examined.

**Conclusion:**

The stronger evidence for the efficacy of melatonergics in prevention of delirium in critically ill patients will be provided for intensive care physicians.

**PROSPERO registration number:**

CRD42019138863.

## 1 Introduction

Delirium is a complex neuropsychiatric syndrome characterized by cognitive impairment and attentional deficits.^[1]^ Delirium in the intensive care unit (ICU) is of high incidence (30%–60%),^[2–4]^ and strongly associated with adverse outcomes, such as increased mortality, prolonged length of stay in intensive care units (ICU-LOS), increased costs, and long-term cognitive sequelae.^[5]^ As increasingly recognized, delirium has turned into one of the most concerned problems for intensive care physicians. Despite delirium in the critically ill appears to be of high incidence and associated with adverse outcome, clinical strategies have been very limited.^[6]^

Melatonin is a neuro-hormone exerting multiple biological effects, mainly in regulating diurnal rhythms, and also in modulating inflammatory as well as immune responses.^[7,8]^ Abundant evidence has indicated that decreasing melatonin levels were linked with delirium.^[9]^ Melatonergics includes melatonin and other melatonin agonists such as ramelteon. Whether supplementation of exogenous melatonin and melatonin agonists could reduce the risk of delirium remains uncertainty. Several randomized controlled studies (RCTs) have been conducted related to the issue. Taking Sultan et al study^[10]^ as an example, 3 hundred elderly patients undergoing hip arthroplasty were randomly assigned to one of four groups; these groups were administrated with melatonin, or midazolam, or clonidine, or no medication, respectively. The results of this study showed that melatonin was associated with significant reduction in incidence of delirium compared with control group as well as the other 2 parallel groups. However, a meta-analysis including four RCTs indicated no significant difference.^[11]^ Since then, several well designed RCTs have been conducted.^[12–15]^ A recent meta-analysis evaluated delirium as a subset but only included three RCTs for this result.^[16]^ With the updated results,^[12–15]^ we aimed to conduct a meta-analysis subjected with the critically ill, in attention to re-evaluating the efficacy of melatonergics in prevention of delirium in ICU.

## 2 Review question

Could melatonergics reduce incidence of delirium in critically ill patients?

## 3 Methods

### 3.1 Study registration

The study has been registered on the PROSPERO (registration number: CRD42019138863) based on the PRISMA-P guidelines.^[17]^

### 3.2 Search methods

Three electric databases (PubMed, Cochrane Library, and Embase) will be searched without language restriction to identify RCTs published from 1960 to April 2019. The reference lists will be searched manually for potentially relevant articles. A search strategy has been developed for the 3 databases as a combination of “melatonin”, “melatonergic”, or “ramelteon” in all fields, and “critically ill”, “critical illness”, “critical care”, or “intensive care” in all fields. The outcome keyword of delirium will not be included in the preliminary screening to avoid missing of potentially relevant references.

### 3.3 Inclusion criteria

#### 3.3.1 Studies

We include studies designed as RCT.

#### 3.3.2 Participants

The study subjects consist of critically ill adults (age ≥18 18 years).

#### 3.3.3 Interventions/Comparators

Melatonergics including melatonin and melatonin agonists are administrated as interventions. The control groups could be no intervention, placebo, or other medication.

#### 3.3.4 Outcomes

The primary outcome measure is incidence of delirium. The secondary outcome is length of stay in ICU (ICU-LOS).

### 3.4 Exclusion criteria

We exclude studies not relevant to ICU. Studies of suboptimal quality (Modified Jadad Score 0-4) will also be excluded.^[18]^

### 3.5 Data collection and analysis

#### 3.5.1 Study screening

The 2 reviewers (ZYB and JZM) will screen the search results according to the title and abstract independently. After the full text obtained, the 2 reviewer will screen the references for potentially relevant studies. The flow chart of selection process will be summarized and reported.

#### 3.5.2 Data extraction

The 2 reviewers (ZYB and JZM) will independently extract data to fulfill a predesigned form including the characteristics of the studies, journal, year of publication, demography and baseline of subjects, intervention and outcomes.

#### 3.5.3 Assessment of study quality

The Cochrane Collaboration's tool will be used to assess selection bias, performance bias, attrition bias and reporting bias.^[19]^ Two reviewers (ZYB and JZM) will independently rate the quality of the RCTs and fulfill the items of risk of bias as low, high, or unclear. Any discrepancies between the 2 reviewers will be solved by a consulting group including two experts (WY and XXM). The quality of evidence resulting from this systematic review was evaluated using the GRADE (Grades of Recommendation Assessment, Development and Evaluation) methodology.^[20,21]^

#### 3.5.4 Statistical analyses and data synthesis

Review Manager, Version 5.3 will be used for data synthesis. The pooled effects of dichotomous outcomes will be analyzed as risk ratio (RR) using Mantel-Haenszel (M-H) technique and 95% confidence intervals (CIs). The pooled effects of continuous outcomes will be analyzed using weighted mean difference (WMD) and 95% CI. A *P* value of less than .05 will be considered to be statistically significant.

#### 3.5.5 Assessment of heterogeneity

*I*^2^ statistic will be used to estimate statistical heterogeneity (*I*^2^ < 30% as low heterogeneity, *I*^2^ of 30% to 70% as medium heterogeneity, *I*^2^ > 70% as high heterogeneity). Clinical heterogeneity will be assessed by the 2 reviewers (ZYB and JZM) and the consulting group (WY and XXM). If high clinical or statistical heterogeneity is observed, a random effect model will be used. Otherwise, a fixed effect model will be chosen.

#### 3.5.6 Subgroup and sensitivity analyses

Subgroup and sensitivity analyses will be conducted to test the robustness of the primary outcome and to further explore the potential influence factors. Subgroups include

(1) differed melatonergics including metalonin and remelteon;
(2) differed age groups including elderly subjects (mean age >60), middle age subjects (mean age 40–60), and younger subjects (mean age <40); and
(3) differed ICU type including surgical ICU, medical ICU, and mixed ICU. Sensitivity analyses will be conducted by excluding any single RCT.

#### 3.5.7 Assessment of publication bias

A funnel plot will be used to assess publication bias when ten or more RCTs are available for quantitative analysis.^[22]^ Egger test will be performed if included studies are less than ten.

#### 3.5.8 Trial sequential analysis

Assessment of the risk of random errors will be done by trial sequential analysis (TSA) (www.ctu.dk/tsa). The results of TSA will determine whether the evidence in our meta-analysis is reliable and conclusive by providing the boundaries of sample size.^[23]^

## 4 Discussion

There have been RCTs^[10,12,24,25]^ suggesting that administration of melatonin and melatonin agonist might be a promising medication in prevention of delirium in at-risk population with the following reasons. First, sleep deprivation is a crucial risk factor of delirium.^[26]^ Exogenous melatonergics have been proved to be apparently associated with improvement of sleep quality and prolongation of sleep duration. Meanwhile, reduction of melatonin levels is associated with development of delirium. Thus, supplementation of exogenous melatonin can remedy disordered melatonin levels.^[27]^ Second, delirium is considered to be caused by inflammation of the central nervous system (CNS). Melatonin is an anti-inflammatory drug and antioxidant functions protectively during the inflammatory response.^[28]^ Third, delirium occurs commonly as a side effect of benzodiazepines, the most commonly used sedatives in ICU.^[29]^ Administration of melatonergics to hypnosis could reduce the dosage of benzodiazepines accordingly. However, the previous meta-analysis showed negative results.^[11]^ With the updated RCTs, the results of this meta-analysis will provide advanced evidence on the efficacy of melatonergics in prevention of delirium in critically ill patients.

## Author contributions

**Conceptualization:** Yibing Zhu, Wei Li.

**Data curation:** Yibing Zhu, Zhiming Jiang.

**Software:** Huibin Huang, Linlin Zhang, Chao Ren.

**Methodology:** Yang Wang, Xiuming Xi, Bin Du.

**Project administration:** Yibing Zhu.

**Supervision:** Wei Li, Xiuming Xi.

**Writing – original draft:** Yibing Zhu.

**Writing – review & editing:** Xiuming Xi, Yongming Yao, Bin Du.
